# Phenotypic and genomic frameworks for precision pharmacotherapy in obesity: a narrative review

**DOI:** 10.3389/fmed.2026.1803474

**Published:** 2026-07-06

**Authors:** Dario S. Lopez Delgado, Miriam Gabriela Reyes-Zermeño, Elian David Sanjuanelo Lemus, Catherine G. Acosta-Celis, María Amparo Kantún-Marín, Martín Gomez-Lujan, Kevin Gabriel Fallaza-Moya, Oscar Muñoz-Chuquilín, Sandra Trujillo-Levano, Giancarlo Gutierrez-Chavez, Cesar Bonilla-Asalde, Oriana Rivera-Lozada, Joshuan J. Barboza

**Affiliations:** 1 Fundación Hospital San Pedro, Pasto, Colombia; 2 Servicio de Gastroenterología, Centro Médico Nacional 20 de Noviembre, ISSSTE, Ciudad de México, Mexico; 3 School of Medicine, Universidad del Norte, Barranquilla, Colombia; 4 Department of Research, Tau Clinical Research Network, Chiclayo, Peru; 5 Department of Nursing, Universidad Autónoma del Carmen, Ciudad del Carmen, Mexico; 6 School of Medicine, Universidad Nacional Federico Villarreal, Lima, Peru; 7 Instituto de Investigaciones Biomédicas (IIBISMED), Cochabamba, Bolivia; 8 Unidad de Postgrado, Universidad Nacional de Trujillo, Trujillo, Peru; 9 Facultad de Ciencias de la Salud, Carrera de Medicina Humana, Universidad Científica del Sur, Lima, Peru; 10 Facultad de Ciencias de la Salud, Universidad Continental, Cusco, Peru; 11 Vice-Rectorate for Research, Universidad Señor de Sipán, Chiclayo, Peru

**Keywords:** GLP-1 receptor agonists, obesity, pharmacogenomics, phenotype-guided treatment, precision pharmacotherapy

## Abstract

**Background:**

Obesity is a chronic, heterogeneous disease with marked interindividual variability in response to anti-obesity medications. Although GLP-1 receptor agonists (GLP-1RAs) and dual incretin agonists produce substantial average weight loss in clinical trials, real-world effectiveness is limited by interindividual variability, gastrointestinal intolerance, incomplete dose escalation, cost and access barriers, poor long-term persistence, and weight plateau or regain in a subset of treated patients. Weight regain is particularly consistent after treatment discontinuation, reinforcing the chronic-care nature of obesity pharmacotherapy.

**Objective:**

To synthesize evidence on phenotypic and genomic determinants of variability in pharmacotherapy response and propose a pragmatic framework for precision pharmacotherapy in obesity.

**Methods:**

Narrative review integrating data from randomized trials, *post-hoc* subgroup analyses, observational cohorts, neurobehavioral studies, pharmacogenomic investigations, and polygenic risk score research addressing predictors of efficacy and tolerability.

**Results:**

Response heterogeneity reflects interactions among central satiety and reward circuitry, baseline metabolic and glycemic status, sex, baseline adiposity, treatment indication, previous GLP-1RA exposure, adherence, and tolerability. Early on-treatment weight change, usually assessed within 12–16 weeks and in some liraglutide studies as early as 1 month, is the most clinically actionable predictor of longer-term outcomes. Phenotype-guided approaches that classify dominant mechanisms such as impaired satiation, postprandial hunger, emotional or hedonic eating, and low energy expenditure offer a feasible bridge between pathophysiology and medication selection. Emerging genomic signals are promising but remain insufficiently replicated for routine clinical use.

**Conclusion:**

Precision pharmacotherapy in obesity is most viable today through phenotype-guided treatment, structured early-response monitoring, and proactive management of adherence and tolerability. Genomic tools may eventually refine treatment selection, but they remain investigational and require prospective, multi-ancestry validation.

## 1 Introduction

Obesity is a chronic, relapsing noncommunicable disease that has significantly increased in prevalence over the past 50 years across nearly all global regions (1). Extensive pooled analyses indicate persistent increases in body mass index distributions and an escalating percentage of adults and children experiencing obesity (2). Obesity is a primary contributor to type 2 diabetes, cardiovascular disease, various malignancies, and premature mortality, significantly impacting worldwide disability-adjusted life years. Contemporary clinical and experimental research characterizes obesity as a biologically heterogeneous condition arising from intricate interactions among genetic predisposition, neuroendocrine regulation, adipose tissue biology, behavior, and environment, rather than merely a result of excessive caloric consumption. Lifestyle modification constitutes the cornerstone of treatment; yet, the sustained maintenance of clinically significant weight loss poses challenges for numerous individuals in standard practice (3). Consequently, professional organizations are progressively endorsing anti-obesity pharmacotherapies, including orlistat, combination treatments, and more recently, glucagon-like peptide 1 receptor agonists and associated incretin-based agents for individuals who fail to attain sufficient weight reduction or risk factor management through lifestyle modifications alone (4). Randomized studies and meta-analyses demonstrate that these pharmacological interventions result in clinically substantial weight reduction and enhancement of cardiometabolic risk factors, but there is considerable variability in individual responses and a propensity for weight rebound following cessation of treatment (5).

Although obesity is now recognized as a heterogeneous disease that includes distinct clinical and metabolic phenotypes, most treatment algorithms still rely mainly on body mass index cut points, the presence of comorbidities and broad contraindications when pharmacotherapy is selected. Phenotypes defined by adipose tissue distribution, ectopic fat deposition, inflammatory status, insulin resistance, eating behavior and other neurobehavioral traits have been described, yet they are rarely used in a systematic way to guide the choice of specific anti-obesity medications in routine care (6). At the same time, advances in human genetics have identified both rare monogenic forms of severe obesity and a highly polygenic architecture underlying common obesity, with many susceptibility loci mapped through large genome wide association studies (7). These discoveries have clarified key biological pathways that regulate body weight, but translation into practical tools that predict individual response or adverse effects with currently available pharmacotherapies remains limited. Existing clinical and pharmacogenetic studies suggest substantial interindividual variability in weight loss and metabolic benefit with agents such as liraglutide and semaglutide, but robust, clinically usable phenotypic or genomic predictors of drug response are still largely absent (8).

Therefore, there is a clear need for a clinically oriented synthesis that integrates phenotypic and genomic information to support a more precise use of anti-obesity medications. In this review, we summarize current evidence on how clinical, metabolic and behavioral phenotypes are associated with the efficacy, tolerability and durability of approved pharmacotherapies for obesity, including incretin-based agents and older drugs. We then examine the contribution of genetic architecture and emerging polygenic approaches to the prediction of treatment response, highlighting where data are promising and where they are inconclusive. Finally, we propose a conceptual framework for precision pharmacotherapy in obesity that links specific phenotypic and genomic profiles to potential treatment pathways and outlines key research priorities needed to move from a trial-and-error approach toward genuinely individualized pharmacological care.

## 2 Methods

### 2.1 Review design and objectives

We conducted a narrative critical review with a focus on integrating and appraising evidence on phenotypic and genomic predictors of response to anti-obesity pharmacotherapy. The primary objective was to synthesize data on how clinical, metabolic and behavioral phenotypes, as well as candidate genes and polygenic scores, modify treatment response and safety profiles of currently approved and emerging pharmacological agents for obesity. Secondary objectives were to identify conceptual and methodological gaps, to evaluate the strength and limitations of the available evidence and to propose a pragmatic precision pharmacotherapy framework for clinical practice and future research.

A formal quantitative meta-analysis was not planned, because of anticipated heterogeneity in study design, phenotypic and genomic definitions, pharmacological regimens and reported outcomes.

### 2.2 Information sources and search strategy

We searched MEDLINE via PubMed, Embase, Scopus and Web of Science Core Collection, from inception to 30 November 2025. Search combined controlled vocabulary terms and free text related to three main domains:

1. Obesity and overweight.
2. Pharmacological treatment of obesity, including glucagon like peptide 1 receptor agonists, dual glucose dependent insulinotropic polypeptide and glucagon like peptide 1 receptor agonists, naltrexone plus bupropion, phentermine plus topiramate, orlistat and other approved or late phase agents.
3. Phenotypic or genomic predictors, including clinical phenotypes, metabolic phenotypes, eating behavior traits, candidate genes, pharmacogenomics, genome wide association studies and polygenic risk scores.

Boolean operators were used to combine terms within and across domains. Reference lists of key trials, systematic reviews and conceptual papers were hand searched to identify additional relevant articles. We also screened major obesity, endocrinology and cardiometabolic journals for recent narrative and conceptual pieces on precision obesity medicine. No language or geographical restrictions were applied; when non-English articles were potentially relevant, English abstracts and translated full texts were considered where available.

### 2.3 Eligibility criteria

We included studies that met the following criteria:

- Population: adults with overweight or obesity, defined according to study specific body mass index or adiposity criteria, from any clinical or community setting.
- Interventions: pharmacological treatments approved or in late development for obesity or obesity related cardiometabolic disease, including incretin-based therapies and combination agents, used for weight management with or without lifestyle interventions.
- Predictors of interest:

 o clinical, metabolic or behavioral phenotypes such as adipose tissue distribution, insulin resistance, metabolic health status, inflammatory markers, appetite and reward related traits, eating behavior and other relevant constructs.
 o genomic or genetic factors, including single variants, candidate genes, pharmacogenomic markers, genome wide association signals and polygenic risk scores related to obesity or cardiometabolic risk.

- Outcomes: measures of treatment response that could be stratified by or modeled in relation to the above predictors. These included percent weight loss, categorical weight loss thresholds, changes in obesity related comorbidities, cardiometabolic risk factors, treatment discontinuation, adverse events and patient reported outcomes when available.

We considered randomized controlled trials, *post-hoc* subgroup and interaction analyses of clinical trials, prospective and retrospective cohort studies, case control studies with pharmacogenomic components, Mendelian randomization studies relevant to drug targets and high quality systematic reviews or meta analyses that provided stratified data or conceptual frameworks on phenotypic or genomic modifiers of response.

We excluded:

- studies focusing exclusively on bariatric surgery or endoscopic procedures without a pharmacological component.
- reports that evaluated pharmacological agents solely for glycemic control in diabetes without weight or obesity related outcomes.
- isolated case reports and small case series without systematic assessment of predictors.
- conference abstracts without sufficient methodological detail.
- narrative pieces that did not present verifiable data or a clearly described selection process.

### 2.4 Study selection and data extraction

Two reviewers independently screened titles and abstracts for potential eligibility, followed by full text assessment of relevant records. Disagreements were resolved through discussion and, when needed, consultation with a third reviewer.

Data extraction was carried out using a structured form that captured: bibliographic information; study design and setting; sample size and population characteristics; type, dose and duration of pharmacotherapy; definition and operationalization of phenotypic or genomic predictors; primary and secondary outcomes; analytical approach to effect modification or stratified analyses; main findings regarding predictors of treatment response and safety; and funding sources and reported conflicts of interest.

For systematic reviews and meta-analyses, we extracted the scope, inclusion criteria and main conclusions, with particular emphasis on any stratified results or conceptual frameworks relevant to precision pharmacotherapy.

### 2.5 Outcomes and domains of interest

The primary outcome was differential response to anti-obesity pharmacotherapy according to phenotypic or genomic characteristics, prioritizing percent weight loss and achievement of predefined weight-loss thresholds when stratified data were available. Secondary domains included changes in glycemic control, blood pressure, lipid profile, and other cardiometabolic markers; improvement or remission of obesity-related comorbidities such as type 2 diabetes, obstructive sleep apnea, and metabolic dysfunction-associated steatotic liver disease; treatment persistence, adherence, discontinuation, and adverse events; and long-term durability of response, including weight regain after treatment discontinuation.

### 2.6 Critical appraisal and assessment of methodological quality

We conducted a design-appropriate qualitative appraisal rather than assigning numeric quality scores. For randomized trials, we assessed randomization, allocation concealment, blinding, follow-up completeness, missing-data handling, and prespecification of subgroup or interaction analyses. For observational studies, we evaluated selection methods, exposure and outcome measurement, confounding control, time-related biases, and sensitivity analyses. For genetic, pharmacogenomic, and polygenic score studies, we additionally considered sample size, statistical power, genotyping quality, ancestry composition, population stratification, multiple-testing correction, replication, score validation, and calibration across ancestry groups. For systematic reviews and guidelines, we assessed the clarity of the question, search comprehensiveness, transparency of study selection and synthesis, and consideration of phenotypic or genomic heterogeneity. Funding sources and conflicts of interest were recorded and considered in the interpretation of findings.

### 2.7 Synthesis and analytical approach

Because of major clinical and methodological heterogeneity in populations, predictors, pharmacological regimens and outcomes, we did not attempt a formal meta-analysis of effect modification. Instead, we organized the evidence into thematic domains that correspond to the structure of the Results section:

1. phenotype-guided pharmacotherapy and clinical or behavioral predictors of response.
2. candidate gene and pharmacogenomic studies.
3. polygenic risk scores and genetically informed subtypes of obesity.
4. integrated precision frameworks that combine phenotypic and genomic information.
5. implementation challenges and equity considerations.

Within each domain, we first summarized the available evidence and then provided a structured critique that focused on internal validity, risk of bias, consistency across studies, mechanistic plausibility and generalizability to diverse clinical settings. Where possible, we highlighted convergent findings from multiple lines of evidence, such as alignment between pharmacogenomic signals and known drug mechanisms, or consistent phenotype response patterns across trials and real-world cohorts.

### 2.8 Reporting

This review did not have a prospectively registered protocol and does not claim adherence to the full methodology of systematic reviews. However, we followed good practice principles for narrative and critical reviews, including explicit definition of objectives, transparent description of information sources and eligibility criteria, independent screening and extraction, and a prespecified thematic synthesis framework. The narrative is structured to align the Methods, Results and Discussion sections so that readers can clearly link the strength of the underlying evidence to each component of the proposed precision pharmacotherapy framework.

## 3 Results

Obesity is not a single biological entity but a heterogeneous syndrome in which individuals with similar body mass index (BMI) can differ markedly in adipose tissue distribution and ectopic fat burden, metabolic and inflammatory status, neurobehavioral drivers of eating, functional limitations, and underlying genetic architecture, differences that plausibly contribute to the substantial interindividual variability observed in pharmacotherapy response and durability (9). This heterogeneity has direct clinical consequences: BMI-based algorithms and comorbidity checklists capture risk incompletely and offer limited guidance on *which* medication is most likely to be effective or tolerated for a given patient profile.

**Figure 1:**
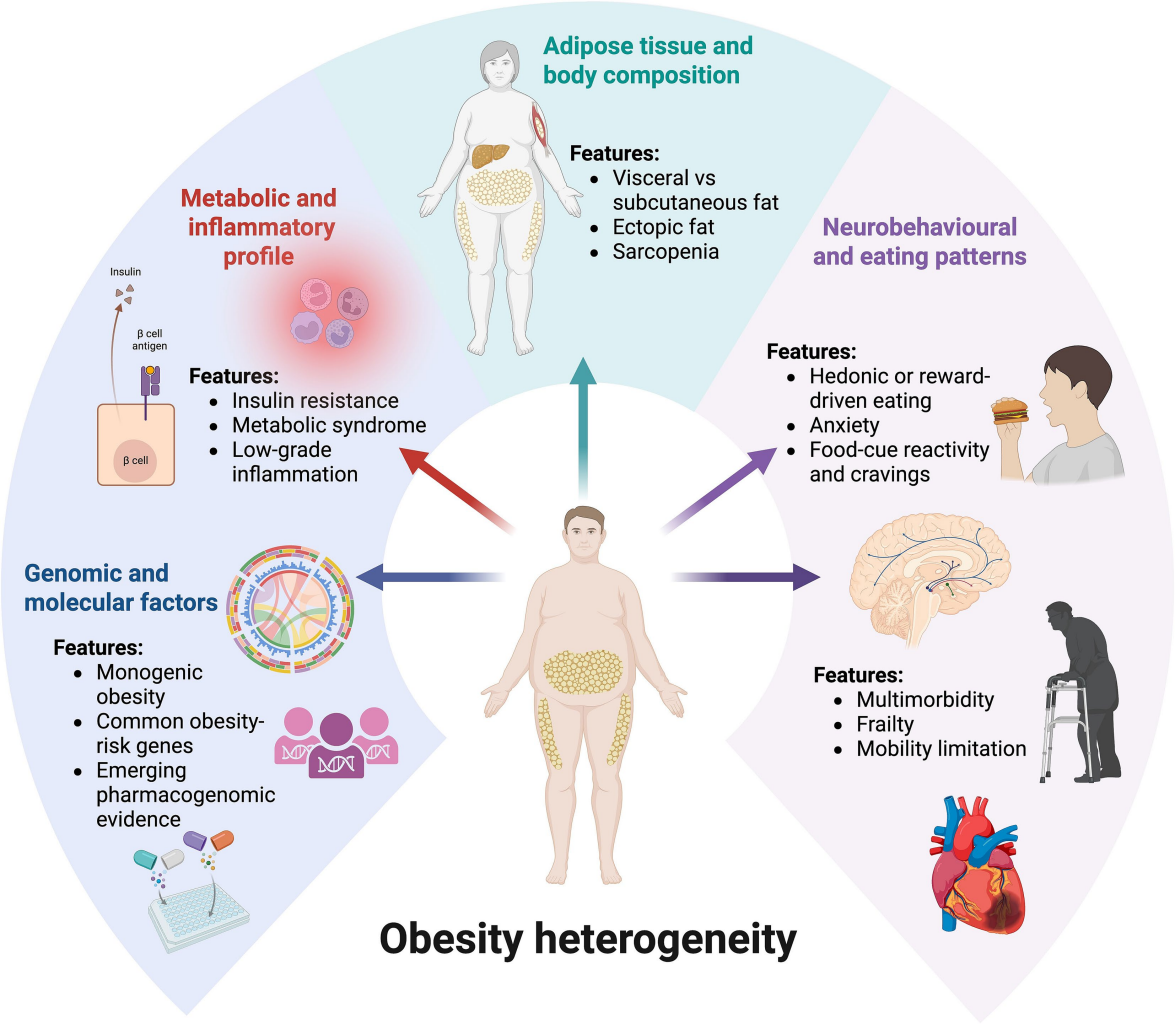
Conceptual framework of obesity heterogeneity informing precision pharmacotherapy. Obesity comprises multiple, partially independent dimensions that may influence clinical risk and treatment response beyond BMI, including (i) adipose tissue distribution and body composition (visceral vs. subcutaneous fat, ectopic fat, sarcopenia), (ii) metabolic–inflammatory profile (insulin resistance, metabolic syndrome, low-grade inflammation), (iii) neurobehavioral and eating patterns (hedonic/reward-driven eating, anxiety, food-cue reactivity and cravings), (iv) genomic and molecular factors (monogenic obesity, common obesity-risk variants, emerging pharmacogenomic signals), and (v) multimorbidity and functional status (frailty, mobility limitation). This figure is intended as a conceptual framework to guide interpretation and identify research priorities; it is not a validated clinical decision algorithm. Created with BioRender.com

Accordingly, we synthesized the evidence through a precision pharmacotherapy lens, organizing results across. Figure 1 provides an orienting map of this framework, summarizing the key axes of obesity heterogeneity.

### 3.1 Current obesity pharmacotherapy and response variability

GLP-1 receptor agonists (GLP-1RAs) and, more recently, dual GLP-1/glucose-dependent insulinotropic polypeptide (GIP) agonists have transformed medical weight management, producing substantial mean weight loss, cardiometabolic benefits, and acceptable safety over 1 to 3 years. These findings support their prioritization in major international guidelines, including recent WHO guidance on GLP-1 therapies for obesity (10).

Liraglutide 3.0 mg was the first GLP-1RA approved for chronic weight management. In SCALE Obesity and Prediabetes, liraglutide produced approximately 8% weight loss versus ~3% with placebo at 56 weeks, with substantially higher proportions achieving ≥5% and ≥10% weight loss (11). In SCALE Diabetes, weight loss was smaller but clinically meaningful and accompanied by HbA1c improvement (12). A 3-year extension showed sustained weight loss and reduced progression to type 2 diabetes in participants with prediabetes (13), while LEADER demonstrated cardiovascular benefit with liraglutide 1.8 mg in high-risk patients with type 2 diabetes (14).

Once-weekly semaglutide 2.4 mg further increased efficacy in the STEP program. In STEP 1, adults with overweight or obesity without diabetes lost about 15% of baseline weight versus 2–3% with placebo, with high proportions achieving ≥10% and ≥15% loss (15). STEP 2 confirmed clinically relevant, although smaller, weight loss in patients with type 2 diabetes (16). Across STEP trials, semaglutide improved glycemic, blood pressure, lipid, and inflammatory markers, with gastrointestinal symptoms as the main adverse events, along with small increases in heart rate, gallbladder events, and caution in patients with advanced diabetic retinopathy and rapid glycemic improvement (17).

Dual incretin agonism with tirzepatide has produced even greater weight reduction. In SURMOUNT-1, tirzepatide 5, 10, and 15 mg weekly led to mean losses of approximately 15, 20, and 21%, respectively, versus about 3% with placebo, with parallel cardiometabolic improvements and reduced progression to type 2 diabetes among participants with prediabetes (18). SURMOUNT-3 and SURMOUNT-4 further emphasized obesity as a chronic treatment condition: adding tirzepatide after intensive lifestyle intervention produced total weight losses approaching 25%, whereas treatment withdrawal led to substantial weight regain and loss of cardiometabolic benefit compared with continued therapy (19, 20).

Other approved anti-obesity medications remain clinically relevant but generally show more modest efficacy or more limiting safety profiles. Naltrexone/bupropion produced mean weight loss of approximately 6% versus 1–2% with placebo in COR-I (21). Phentermine/topiramate controlled-release achieved mean weight reductions near 10% in CONQUER, with improvements in cardiometabolic risk factors but important concerns related to paresthesias, cognitive or mood symptoms, and teratogenicity (22). Orlistat has lower efficacy but long-term evidence from XENDOS, where it produced modest additional weight loss and reduced incident type 2 diabetes, although gastrointestinal adverse effects and fat-soluble vitamin loss frequently limited adherence (23).

Across pivotal trials, a consistent finding is wide interindividual variability in response. Even with semaglutide 2.4 mg or high-dose tirzepatide, some patients fail to achieve 5–10% weight loss, whereas others exceed 20% (16, 17, 19). Diabetes status, baseline weight, cardiometabolic comorbidities, concomitant medications, lifestyle co-interventions, and early response at 3 to 4 months all influence treatment outcomes. Tolerability also varies substantially, affecting discontinuation and real-world effectiveness (24). These patterns support the view, reflected in contemporary guidelines, that GLP-1RAs and dual agonists should be used as long-term, individualized, disease-modifying therapies rather than short-term weight-loss drugs (4). From a precision pharmacotherapy perspective, this evidence defines the average benefit and safety of each agent while highlighting the need to move toward phenotype-guided and, eventually, biomarker-guided medication selection. Table 1 summarizes the key clinical trials of weight-loss pharmacotherapy.

**Table 1:** Summary of pivotal randomized trials of current obesity pharmacotherapies.

Drug / program	Trial (population)	N / duration	Mean Δ weight vs placebo (% BW)	Responders ≥5% / ≥10% / ≥15%	Main adverse events
Liraglutide 3.0 mg	SCALE Obesity & Prediabetes (no T2D) (11)	3,731/56 wks.	≈8% vs. ≈ 3%	≈63% / ≈33% / ~14%	GI (nausea, vomiting, diarrhea), gallbladder events
Liraglutide 3.0 mg	SCALE Diabetes (T2D) (12)	846/56 wks.	≈6% vs. ≈ 2%	54% / 25% / NR	GI events, slight ↑HR, rare pancreatitis signal
Semaglutide 2.4 mg	STEP 1 (obesity/overweight, no T2D) (15)	1961/68 wks.	14.9% vs. 2.4%	~86% / ~69% / ~51%	GI events, gallbladder disease, mild ↑HR
Semaglutide 2.4 mg	STEP 2 (overweight/obesity + T2D) (16)	1,210/68 wks.	9.6% vs. 3.4%	68.8% / lower ≥10% vs. STEP 1	GI events more frequent than placebo
Tirzepatide 5–15 mg	SURMOUNT-1 (obesity/overweight, no T2D) (18)	2,539/72 wks.	~15–21% vs. ~ 3%	majority ≥5%, up to ~60% ≥ 20%	GI (nausea, diarrhea, constipation), mild ↑HR
Tirzepatide	SURMOUNT-3 (after lifestyle run-in) (19)	806/72 wks.	~21% additional vs. weight regain	very high ≥5%, many ≥15–20%	Similar GI profile, some AE-related discontinuation
Tirzepatide	SURMOUNT-4 (withdrawal, obesity, no T2D) (20)	670/88 wks.	≈ − 25% WL with continued TX vs. regain	most maintain ≥5%, many ≥15–20%	GI events, weight and risk-factor rebound when treatment is stopped
Naltrexone/bupropion ER	COR-I (overweight/obesity ± comorbidities) (21)	1742/56 wks.	6.1% vs. 1.3%	48% / 25% / NR	Nausea, constipation, headache, insomnia, ↑BP/HR, seizure risk
Phentermine/topiramate ER	CONQUER (overweight/obesity + ≥ 2 comorbidities) (22)	2,487/56 wks.	≈10% vs. ≈ 1–2%	majority ≥5%, ~50% ≥ 10%	Paresthesias, cognitive/mood effects, dry mouth, insomnia, teratogenicity
Orlistat	XENDOS (obesity, long-term diabetes prevention) (23)	3,305/4 yrs	≈2–3% > placebo	modest ↑ ≥ 5%, few ≥10%	Steatorrhea, fecal urgency, oily stools, fat-soluble vitamin loss

Δ, change; BW, body weight; WL, weight loss; T2D, type 2 diabetes; BP, blood pressure; SBP, systolic blood pressure; TG, triglycerides; GI, gastrointestinal; HR, heart rate; ER, extended-release; QoL, quality of life; NR, not reported; TX, treatment; AE, adverse event.

### 3.2 Phenotype-guided pharmacotherapy: clinical and behavioral predictors

Obesity phenotypes capture the observable expression of biological, behavioral, and metabolic mechanisms that contribute to excess body weight and help explain interindividual variability in pathophysiology, etiology, metabolic behavior, and treatment response (25, 26). Table 2 summarizes representative evidence supporting each phenotype and the predominant evidence type underlying the proposed phenotype-guided framework.

**Table 2:** Evidence map supporting the key obesity phenotypes used in the phenotype-guided pharmacotherapy framework.

Phenotype	Representative primary evidence supporting the phenotype construct/measurement	Representative evidence informing clinical translation / treatment matching	Predominant evidence type for the phenotype-guided framework
Hungry brain (abnormal satiation; larger meal size / higher “calories to satiation”)	Physiologic variability in calories-to-satiation measured by ad libitum meal testing, with substantial inter-individual heterogeneity in adults with obesity (48).	Pragmatic, real-world randomized trial demonstrating superior 12-month weight loss with phenotype-guided medication selection vs. non-guided care (includes hungry brain phenotype) (6). Randomized evidence that calories-to-satiation strata predict differential response (greater benefit with phentermine/topiramate when calories-to-satiation is high) (48). Neuroimaging reveals persistent activation in reward regions even after eating (96, 97), while genetic studies implicate central nervous system pathways regulating satiety (98)	Randomized clinical evidence (pragmatic RCT + mechanistic RCT), supported by physiologic observational measurement studies
Hungry gut (abnormal satiety; reduced post-meal satiety often linked to faster gastric emptying)	Cross-sectional clinical physiology data linking gastric emptying metrics with postprandial appetite/satiety sensations in obesity (99).	Pragmatic phenotype-guided randomized trial (includes hungry gut phenotype and phenotype-guided medication choice) (6). Pharmacodynamic randomized study targeting accelerated gastric emptying with GLP-1–based therapy (6). Placebo-controlled randomized trial showing liraglutide effects on gastric emptying and weight outcomes in obesity (100). Altered gut hormone profiles (e.g., GLP-1) are implicated (101), with gut-brain axis dysfunction supported by microbiome research (102)	Pragmatic RCT + mechanistic RCTs, supported by observational physiology studies
Emotional hunger (hedonic/emotional eating; reward-driven intake, cravings, loss of control)	Behavioral/psychological phenotyping studies showing emotional eating as a measurable domain in weight-loss interventions (103).	Pragmatic phenotype-guided randomized trial (includes emotional hunger phenotype and matching to anti-obesity medications) (6). Phase 3 randomized trial of naltrexone/bupropion demonstrating clinically meaningful weight loss vs. placebo (relevant to reward/craving pathway targeting) (21). Large clinical sample showing early improvements in cravings are associated with longer-term weight loss success (104). Cross-sectional study otes the potential benefits of addressing impulsivity and reward-based eating for the hedonic Impulsive phenotype (105).	Randomized trial evidence for pharmacotherapy efficacy, supported by behavioral observational evidence for the phenotype construct
Slow burn (decreased metabolic rate; low energy expenditure / adaptive thermogenesis)	Prospective evidence that lower energy expenditure predicts future weight gain (risk phenotype for “low burn”) (106). Prospective cohort data supporting resting metabolic rate as a predictor of weight change trajectories (107). Long-term follow-up showing persistent metabolic adaptation after major weight loss.	Pragmatic phenotype-guided randomized trial includes “slow burn” (decreased metabolic rate) phenotype in the classification and medication selection framework (6). Biomarker studies show distinct metabolomic signatures in this group (108)	Strong observational/physiology evidence for the construct + pragmatic RCT evidence supporting phenotype-guided strategy as a whole

The table summarizes (i) representative primary evidence underpinning each phenotype’s construct and measurement, (ii) representative evidence informing clinical translation and treatment matching, and (iii) the predominant evidence type supporting the phenotype-guided approach.

These phenotypes reflect heterogeneity across interconnected pathways, including neuroendocrine regulation of appetite, energy expenditure, and adipogenesis mediated by leptin, insulin, ghrelin, GLP-1, and PYY (27, 28). Genetic variants affecting hypothalamic and dopaminergic signaling may predispose individuals to hyperphagia, reduced satiety, and altered reward processing (29, 30). Behavioral phenotypes include impulsive, emotional, and hedonic eating patterns linked to reward- and stress-related neural circuits (31, 32), whereas metabolic phenotypes arise from interactions among diet, environment, lifestyle, gut microbiota, and genetic susceptibility that influence insulin sensitivity, energy utilization, and fat storage (33, 34).

Acosta and colleagues proposed a pragmatic four-phenotype model, supported by randomized comparisons of standard obesity care versus phenotype-guided pharmacotherapy across two independent cohorts, to identify clinically meaningful drivers of obesity (6). The hungry brain phenotype reflects impaired satiation and higher caloric intake needed to feel full; the hungry gut phenotype reflects reduced postprandial satiety and earlier return of hunger; emotional hunger captures hedonic or emotion-driven eating; and the slow-burning phenotype refers to low basal energy expenditure or metabolic adaptation to calorie restriction. These categories should be interpreted as dominant and potentially overlapping mechanisms rather than mutually exclusive types, because mixed phenotypes are common and may change over time with weight loss, behavioral interventions, and medication effects.

#### 3.2.1 Predictors of early response (weight loss at 12–16 weeks of GLP-1 therapy) and implications for decision to continue or change medication

Emerging real-world data and *post-hoc* analyses suggest that early weight loss after initiation of GLP-1RA therapy is one of the most practical predictors of longer-term response. In a retrospective cohort of 209 patients treated with liraglutide, greater weight loss at 3 months significantly predicted achievement of ≥5% weight reduction at 6 months, even after adjustment for age, sex, baseline HbA1c, and other metabolic parameters; lower baseline BMI was also associated with a higher likelihood of significant weight loss (35). Maccora et al. found that weight loss at 1 month was the only predictor of subsequent treatment response, with ≥5% weight loss achieved by 27, 45, and 57% of patients at 1, 3, and 6 months, respectively (36). This “early response predicts later success” pattern is consistent with pediatric and adolescent GLP-1RA studies, in which BMI reduction by approximately 12–16 weeks correlated with greater long-term weight loss (37).

Clinically, these findings support reassessment at approximately 12–16 weeks as a structured decision point for continuing, intensifying, or switching therapy. However, thresholds differ across medication classes. The FDA label for liraglutide 3.0 mg recommends discontinuation if <4% baseline weight loss is achieved by week 16, whereas naltrexone/bupropion recommends discontinuation if <5% weight loss is achieved after 12 weeks on the maintenance dose (38). For phentermine/topiramate, response is assessed after 12 weeks at 7.5/46 mg, with escalation if <3% weight loss is achieved, and reassessed after 12 weeks at 15/92 mg, with discontinuation if <5% weight loss is achieved (39). Continuation decisions may also consider early cardiometabolic and symptom-level improvements, particularly when glycemic control or cardiovascular risk reduction is a major treatment goal. In pooled SCALE analyses, early responders to liraglutide achieved greater 1-year weight loss and larger improvements in cardiometabolic markers and quality-of-life measures (12). Because delayed responders may occur, early nonresponse should prompt assessment of adherence, dose titration, tolerability, and lifestyle implementation before concluding true treatment failure, especially in patients with severe insulin resistance or long-standing obesity (40, 41).

Beyond early weight trajectory, several baseline clinical and behavioral factors may influence GLP-1RA response. Evidence on baseline adiposity is mixed: one diabetes cohort found greater relative weight loss among patients with lower baseline BMI, whereas another 52-week observational study found that responders had higher baseline BMI, greater fat mass, and lower muscle mass (35). Sex may also be relevant, as a real-world analysis of patients treated with semaglutide, liraglutide and tizepatide found that women were more likely to be hyper-responders, although other variables, including age, baseline BMI, glycemic status, depression, anxiety, and sedentary behavior, were not consistently associated with response (41–44). Trial data suggest that individuals without diabetes may have higher early-response rates than those with diabetes (45), while younger age and shorter disease duration have also been associated with better 1-year response in some cohorts (46). Finally, appetite-related phenotypes may contribute to response heterogeneity: slower gastric emptying and lower caloric intake during an ad libitum meal were associated with greater liraglutide-induced weight loss (47), and preliminary precision pharmacotherapy data suggest that satiation-related phenotypes or genetic scores may help identify patients more likely to respond to liraglutide versus appetite-suppressing therapies (48).

### 3.3 Genomic predictors of drug response: candidate genes and pharmacogenomics

GLP-1RAs act through peripheral and central pathways that regulate glucose homeostasis, appetite, satiety, reward processing, and energy balance, which may partly explain interindividual variability in clinical response (49). Emerging pharmacogenomic findings can be interpreted both as predictors of response magnitude, including weight-loss trajectory and cardiometabolic change, and as mechanistic modifiers that identify biological pathways involved in response or resistance. For example, GLP1R variants may influence gastric emptying, insulin secretion, and central satiety signaling, whereas variants affecting dopaminergic or reward-related pathways may be more relevant for therapies targeting craving or hedonic eating. However, current effect sizes, replication, and clinical validity remain insufficient for routine implementation (50).

Early clinical investigations in patients with type 2 diabetes showed marked interindividual differences in GLP-1–mediated glucose-lowering effects, even under standardized experimental conditions, suggesting that pretreatment metabolic characteristics such as fasting glycemia may influence therapeutic benefit (51). Human neuroimaging studies further support a central mechanism, showing that GLP-1 receptor activation modulates brain regions involved in appetite and reward, including the insula, amygdala, orbitofrontal cortex, ventral tegmental area, and nucleus accumbens, reducing food cue-related neural activity, food intake, and the hedonic value of palatable foods (52, 53). These findings support the hypothesis that variability in central reward and satiety circuitry may contribute to differential response to GLP-1–based therapies.

Although GLP-1RAs produce clinically meaningful average weight loss, response distributions are wide. Liraglutide and broader GLP-1RA data show that some patients fail to achieve predefined therapeutic thresholds despite treatment exposure, indicating that non-response is a clinically relevant feature rather than an exception (54, 55). Therefore, GLP-1RA effectiveness should not be interpreted as a uniform class effect, but as the result of interactions among central nervous system signaling, peripheral metabolic status, adherence, tolerability, and individual biological predisposition (56). A notable example of a drug-response genomic signal is the emerging association between NBEA and differential weight-loss response to GLP-1RAs, discussed in Section 3.4.1.

#### 3.3.1 Genes involved in response to topiramate or other AOMs (INSR, HNF1A, MC4R in monogenic obesity)

Genetic variation contributes to the heterogeneous response to anti-obesity medications, involving pathways related to metabolism and appetite regulation beyond GLP-1 signaling. Early evidence in this field comes from candidate-gene studies evaluating topiramate, which identified associations between weight-loss response and variants in genes related to insulin signaling, including INSR and HNF1A. These findings suggest that individual differences in glucose–insulin regulatory pathways may influence the effectiveness of certain AOMs (57).

Converging evidence highlights the melanocortin-4 receptor (MC4R) as a key genetic determinant of obesity risk and treatment response. Large meta-analyses demonstrate that common variants such as rs17782313 are consistently associated with increased obesity susceptibility, underscoring the central role of MC4R, in appetite regulation and energy balance (58). Further analyses incorporating multiple.

MC4R polymorphisms and haplotypes indicate that gene–environment interactions within this locus contribute to interindividual variability in obesity phenotypes (59).

At the extreme end of the genetic spectrum, rare and monogenic forms of obesity caused by pathogenic variants in MC4R and related leptin–melanocortin pathway genes are characterized by severe, early-onset obesity and reduced responsiveness to conventional pharmacological interventions. This body of evidence reinforces the concept that underlying genetic architecture can shape both obesity pathogenesis and therapeutic response, highlighting the potential value of genetically informed treatment selection (60).

#### 3.3.2 Stability and clinical significance of GLP1R results

The most frequently examined GLP1R variant, rs6923761 (Gly168Ser), has shown endpoint-dependent associations with GLP-1RA response. For glycemic outcomes, evidence is relatively consistent: the A/Ser allele has been associated with a smaller HbA1c reduction during GLP-1RA therapy. In a multi-cohort meta-analysis, Dawed et al. reported a 0.08% smaller HbA1c reduction per Ser allele (*p* = 6.0 × 10^-5) (61). Functional studies support the biological plausibility of this association, showing that GLP1R missense variants may reduce cAMP production and impair *β*-cell insulin secretion *in vitro* (62). Similarly, in Chinese patients with type 2 diabetes, the rs3765467 A allele was associated with a smaller HbA1c reduction compared with the GG genotype (*p* = 0.002) (63).

By contrast, associations between GLP1R variation and weight loss are less consistent. Smaller cohort studies have suggested that the rs6923761 A allele may be associated with greater weight reduction, including a larger absolute weight change, accelerated weight-loss kinetics, or allele-dose-dependent effects (64–66). However, this apparent weight-loss advantage has not consistently translated into broader metabolic benefit, and larger or more pragmatic studies have generally failed to confirm a significant association with weight change, including analyses by German et al., Maselli et al., and Candido et al. (66–68). Overall, the available data suggest that GLP1R variants may have more robust relevance for glycemic response than for weight-loss prediction.

ARRB1, which encodes *β*-arrestin 1, has emerged as a promising genomic signal for glycemic response to GLP-1RA therapy. The low-frequency rs140226575 variant (Thr370Met) was identified in a GWAS meta-analysis and subsequently replicated in an independent randomized trial cohort, where the Met allele was associated with a 0.25% greater HbA1c reduction (61). Because this association was not observed with other glucose-lowering therapies, it may reflect specificity for the GLP-1 signaling pathway. Although the individual effect size is modest, combined favorable ARRB1 and GLP1R genotypes identified a small subgroup, approximately 4% of the population, with a substantially greater HbA1c response and delayed treatment failure (61, 69). The favorable ARRB1 allele also appears more frequent in Hispanic and American Indian/Alaska Native populations than in White European populations, highlighting the importance of ancestry-diverse pharmacogenomic research (61, 69).

Despite these signals, the clinical utility of GLP1R and ARRB1 testing remains limited. Evidence for weight-loss prediction is inconsistent, several studies are underpowered, and most findings require prospective validation in larger and more diverse populations (61, 70). Therefore, routine GLP1R or ARRB1 genetic testing cannot currently be recommended to guide anti-obesity pharmacotherapy. Future studies should prioritize adequately powered, multi-ancestry cohorts with standardized response definitions, prespecified pharmacogenomic analyses, and independent replication before these biomarkers can be integrated into precision obesity care (66, 71).

### 3.4 Polygenic risk scores

Polygenic risk scores (PRS) have been increasingly investigated to predict therapeutic response to different obesity management strategies, particularly regarding bariatric surgery (72). In a cohort of 793 participants undergoing biliopancreatic diversion with duodenal switch monitored over a period of 48 months, the inclusion of a PRS constructed from 186 BMI-associated single nucleotide polymorphisms (SNPs) to a prediction model that included initial BMI, age, sex and surgery modality with an area under the curve (AUC) of 0.867, significantly increased its accuracy, raising the AUC by 0.021 (95% CI 0.005–0.038), however the inclusion of a simplified PRS constructed from 11 BMI-SNPs did not significantly alter the AUC of the initial model evaluated (73). A 5-year cohort in Spain with 104 participants analyzed the outcomes following Roux-en-Y gastric bypass and sleeve gastrectomy revealed that patients with lower PRS (7 SNPs) achieved greater total weight loss at 24 and 60 months compared to those with higher PRS, similarly other 5-year cohort in Spain with 106 participants following bariatric surgery found better outcomes in patients with lower scores in a 50 SNPs PRS (74, 75).

Regarding pharmacological interventions, clinical data from 1,055 American adults with type 2 diabetes demonstrated that individuals with intermediate and high BMI PRS (built from 953,416 SNPs) experienced significantly less weight loss (0.7 and 1.5% respectively) over 52 weeks of GLP-1RA therapy compared to the low PRS group and individual SNPs were not significantly correlated with weight loss (76). The results derived from these studies suggest that SNPs that predispose adults to obesity also modify the efficacy of pharmacological and surgical interventions (75, 76). However, variety in these associations is observed, certain composite genetic risk scores failed to demonstrate a significant predictive capacity for weight loss or regain in some cohorts with bariatric participants (77).

The integration of genetic profiling into clinical prediction models is proposed to enhance the personalization of obesity treatment, although the added predictive value varies across different study designs (72, 73, 77). Limitations restrict the translation of PRS into routine clinical practice. An important problem is the ancestry bias, as the majority of PRS are derived from genome-wide association studies conducted in European populations, thereby limiting their validity and utility in non-European ancestry groups (72, 76). Additionally, statistical power is compromised by small sample sizes in bariatric cohorts, which hinders the ability to detect small effect sizes which are typical of common genetic variants (78, 79). The focus on candidate gene approaches in some cohorts, rather than wide genome-wide scans, may have introduced selection bias and excluded other relevant genetic contributors (79, 80). Furthermore, the complex interactions between SNPs and environmental factors, such as diet and lifestyle, are not fully considered in current prediction models and the cost-effectiveness of implementing these genetic screenings requires further evaluation (72).

#### 3.4.1 Neurobeachin (NBEA) and its genetic score for “non-responders” to liraglutide/GLP-1

Building on the mechanistic framework outlined above for GLP-1–based therapies, recent work has moved toward drug-specific genomic stratification of treatment response. In this context, NBEA has emerged as a candidate locus of interest, particularly for identifying patients with attenuated weight-loss response to liraglutide and related GLP-1 receptor agonists. This aligns with the precision pharmacotherapy paradigm, which proposes that inter-individual variability in treatment response should be addressed through patient stratification rather than dose augmentation or treatment prolongation. Accordingly, the integration of clinical phenotypes with molecular and genetic information is increasingly considered a necessary step for optimizing therapeutic choice in metabolic diseases (81).

The study by Mariam-Smith et al. provides the first robust genomic evidence linking NBEA to differential weight-loss response to GLP-1 RAs. Leveraging large real-world datasets from the NIH All of Us program and independent replication in the UK Biobank, the authors identify NBEA-associated variants and construct a genetic score that stratifies individuals according to treatment responsiveness. Importantly, carriers of higher genetic risk exhibit consistently attenuated weight loss, particularly among liraglutide users, independent of baseline BMI, glycemic status, or other clinical predictors, highlighting a genetically driven non-responder phenotype (82).

Previous experimental work has linked NBEA to regulation of feeding behavior and body weight through its role in neuronal protein trafficking and synaptic organization (83). At the molecular level, NBEA functions as a protein kinase A anchoring protein, supporting its involvement in cAMP/PKA signaling pathways central to GLP-1–mediated satiety effects (84).

The main biological, clinical, and genetic determinants contributing to interindividual variability in response to GLP-1 RAs are summarized in Table 3.

**Table 3:** The main biological, clinical, and genetic determinants contributing to interindividual variability in response to GLP-1 receptor agonists (GLP-1RAs) are summarized.

Domain	Factor	Biological/Clinical relevance	Key references
Central nervous system	Hypothalamic satiety signaling	GLP-1R activation modulates appetite control and energy balance through central pathways influencing satiety	(44)
Central nervous system	Reward-related brain circuits (insula, amygdala, OFC)	GLP-1R agonists reduce food-cue–related neural activation and caloric intake, contributing to heterogeneous behavioral responses	(47)
Metabolic status	Baseline glycemia	Pretreatment fasting glucose influences magnitude of glucose-lowering response to GLP-1	(46)
Clinical response	Weight-loss variability	Liraglutide induces highly variable weight loss, with a subset of patients achieving limited or no benefit	(50, 51)
Treatment framework	One-size-fits-all limitation	Interactions between CNS signaling, peripheral metabolism, and patient factors limit uniform therapeutic efficacy	(52)
Precision medicine	Patient stratification	Integrating clinical phenotypes with molecular and genetic data may optimize treatment selection	(77)
Genetics (core)	NBEA genetic score	NBEA-associated variants identify genetically driven non-responders to GLP-1RA, particularly liraglutide	(78)
Genetics (mechanistic)	NBEA neuronal function	NBEA regulates synaptic organization and neuronal protein trafficking relevant to feeding behavior	(79)
Molecular signaling	cAMP/PKA pathway	NBEA acts as a PKA anchoring protein, supporting a role in GLP-1–mediated central satiety signaling	(80)

cAMP, cyclic adenosine monophosphate; CNS, central nervous system; GLP-1, glucagon-like peptide-1; GLP-1R, glucagon-like peptide-1 receptor; GLP-1RA, glucagon-like peptide-1 receptor agonist; OFC, orbitofrontal cortex; PKA, protein kinase A; NBEA, neurobeachin.

### 3.5 Integrated precision algorithm for obesity management

We propose a conceptual algorithm that integrates standard clinical assessment, phenotype-based stratification, and, when available, exploratory genetic risk profiling to support individualized anti-obesity pharmacotherapy. This framework should be interpreted as a hypothesis-generating clinical model rather than a validated decision rule, because direct evidence supporting algorithm-guided treatment selection remains limited and requires prospective validation.

#### 3.5.1 Step 1: initial clinical and genomic assessment (patient profile)

The objective is to stratify risk not only by current adiposity, but also by biological predisposition and obesity-related complications.

(1) Standard clinical data: (i) Measure BMI and waist circumference; Systematically identify key comorbidities—prediabetes/type 2 diabetes (T2D), cardiovascular disease, obstructive sleep apnea (OSA), and hepatic steatosis (MASLD/MASH)—because they directly influence therapeutic priorities (85).
(2) Genomic evaluation and PRS: (i) Use a BMI PRS to estimate genetic susceptibility to severe obesity and a higher likelihood of resistance to weight loss with lifestyle interventions alone (76).(ii) Targeted variant review: Consider variants in appetite and reward-related pathways (e.g., MC4R and dopaminergic genes such as DRD2/DRD4), which may help explain hyperphagia or addiction-like eating behaviors (82).

PRS information can help anticipate who may need earlier intensification and who may be at higher risk of weight regain.

#### 3.5.2 Step 2: phenotypic classification (the “engine” of obesity)

Classify patients according to the predominant physiological mechanism driving weight gain:

Phenotype A: Hungry Brain (abnormal satiety) needs large amounts of food to feel full.

Phenotype B: Emotional/Hedonic hunger—eating driven by cravings, stress, or reward.

Phenotype C: Hungry Gut (rapid gastric emptying/low postprandial satiety) hunger shortly after meals.

Phenotype D: Slow metabolism (low energy expenditure) reduced metabolic rate.

#### 3.5.3 Step 3: selecting targeted pharmacotherapy (precision pharmacotherapy)

Choose therapy based on the intersection between comorbidities (Step 1) and phenotype (Step 2).

##### 3.5.3.1 Option a: GLP-1 RAs and dual agonists (semaglutide /tirzepatide/liraglutide)

Primary indication: “Hungry Gut” and “Hungry Brain” phenotypes, and patients with T2D and/or high cardiovascular risk (19).

Specific selection:

- Tirzepatide: Prefer when the goal is maximal weight reduction (up to ~20.9%) (18), or when OSA, HFpEF, or hepatic steatosis co-exist (85).
- Semaglutide: Prefer for substantial weight loss (~15%), particularly when cardiovascular risk reduction (MACE) is a priority or when comorbidities such as knee osteoarthritis are prominent (17).
- Liraglutide: A proven option (~8%), especially when access to higher-potency agents is limited or when phenotypes suggest rapid gastric emptying (14).

Genomics: Certain GLP-1 receptor (GLP1R) variants may be associated with better response and can help calibrate expectations (Explained in section 3.2).

##### 3.5.3.2 Option B: naltrexone/bupropion

Primary indication: Emotional/hedonic hunger or an “Hungry brain” phenotype (81).

Genomics: Particularly relevant when genetic testing (e.g., GARS) suggests dysfunction/hypodopaminergic traits, supporting a mechanistic rationale for craving control.

##### 3.5.3.3 Option C: phentermine/topiramate

Primary indication: “Hungry Brain” phenotype when reduction of pronounced hyperphagia is a central objective (2).

##### 3.5.3.4 Special annotations

Although the therapeutic options described above are primarily mapped to specific obesity phenotypes, it is important to recognize that: pleiotropic effects and clinical overlap between phenotypes are common; for example, a patient may meet criteria for both hungry brain and emotional hunger, and drivers may evolve during treatment. In such cases, prioritize the predominant driver at the current timepoint and reassess at each checkpoint.

Hungry Brain phenotype: This Patients may benefit from CNS-acting agents such as phentermine/topiramate, which directly suppress appetite. However, it should be noted that incretin-based therapies also exert central effects on hypothalamic and brainstem satiety circuits, making GLP-1 RAs a rational option either as monotherapy or in sequential treatment strategies (51).

Emotional/hedonic hunger phenotype: naltrexone/bupropion is particularly suited due to its modulation of dopaminergic and opioid reward pathways. Emerging evidence suggests that central GLP-1 signaling may also influence reward-related brain regions, indicating a potential complementary role for incretin-based therapies in selected patients with overlapping phenotypes (53).

Slow metabolism/low energy expenditure phenotype: Although no current anti-obesity medication exclusively targets reduced basal metabolic rate, incretin-based therapies may still confer benefit through caloric intake reduction and improved metabolic efficiency, particularly when low energy expenditure coexists with impaired satiety or insulin resistance. In such cases, pharmacologic therapy should be combined with structured resistance training and metabolic optimization (86).

#### 3.5.4 Step 4: early response monitoring and adjustment

This approach requires early reassessment to minimize therapeutic inertia (47, 54):

- Early responder rule: Evaluate weight loss at 12–16 weeks (3–4 months) (2).
- Adequate response: ≥5% weight loss (or ≥3% in diabetes) → continue (2).
- Non-response: <5% → discontinue and switch to an alternative mechanism, escalate to combination therapy, and/or consider surgery when appropriate, because durable response is less likely (2).

Genetic adjustment: A high obesity PRS may indicate stronger biological “defense” of body weight and can justify earlier intensification and long-term maintenance planning.

**Figure 2:**
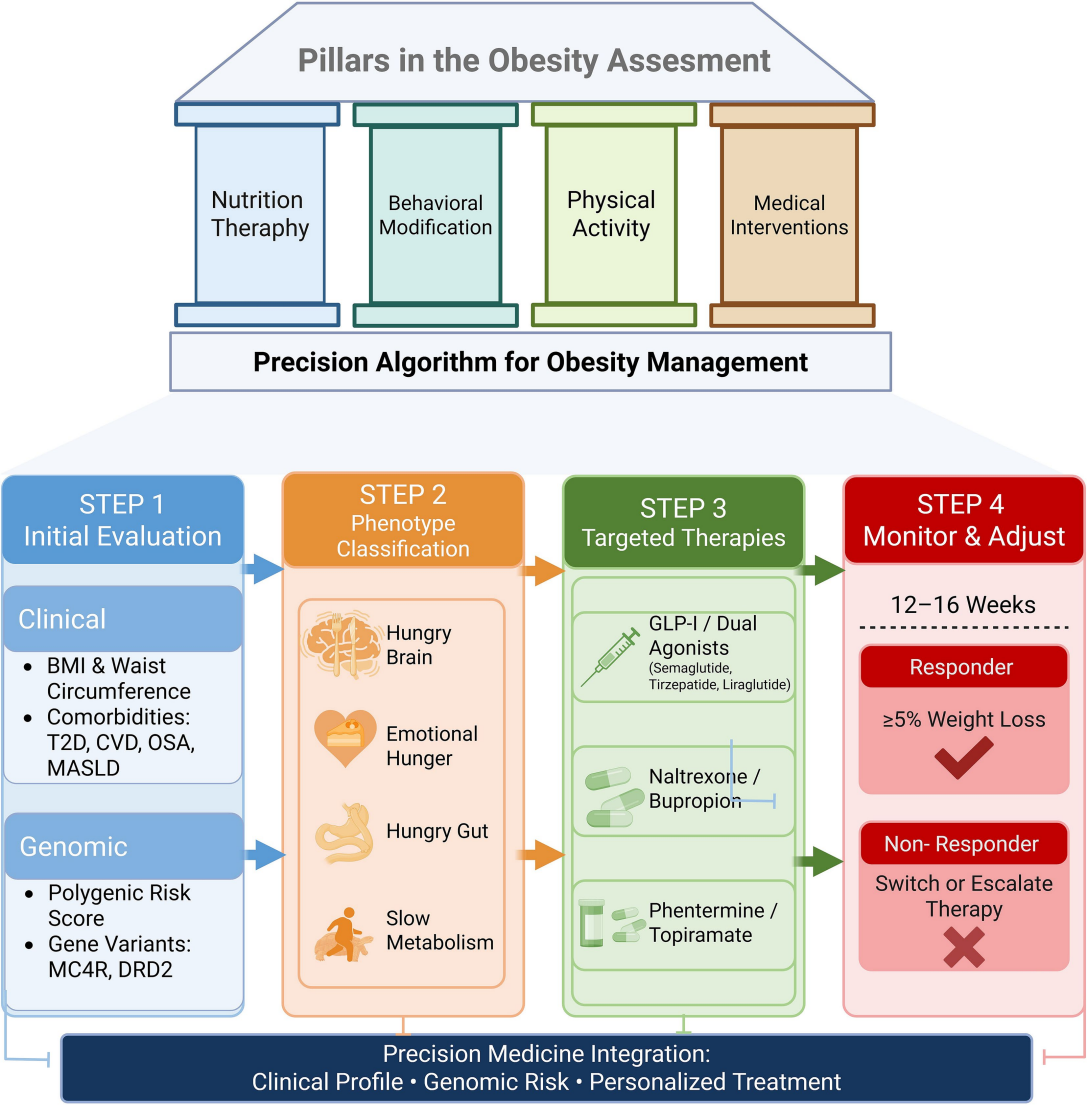
Precision algorithm for obesity management, integrating lifestyle pillars and precision pharmacotherapy assessment. The framework summarizes four pillars of obesity care (nutrition therapy, behavioral modification, physical activity, and medical interventions) and a stepwise approach: Step 1, initial evaluation of clinical adiposity measures and obesity-related comorbidities plus genomic risk profiling; Step 2, phenotype classification (hungry brain, emotional hunger, hungry gut, slow metabolism) to capture predominant drivers of weight gain; Step 3, selection of targeted pharmacotherapies (GLP-1–based or dual incretin agonists, naltrexone/bupropion, or phentermine/topiramate); and Step 4, reassessment at 12–16 weeks to monitor response (≥5% weight loss) and adjust therapy (switch or escalate) in non-responders, integrating clinical profile and genomic risk to personalize treatment. This figure is intended as a conceptual framework to guide interpretation and identify research priorities; it is not a validated clinical decision algorithm. Abbreviations: BMI, body mass index; CVD, cardiovascular disease; DRD2, dopamine receptor D2 gene; GIP, glucose-dependent insulinotropic polypeptide; GLP-1, glucagon-like peptide-1; MASLD, metabolic dysfunction–associated steatosis liver disease; MC4R, melanocortin 4 receptor gene; OSA, obstructive sleep apnea; PRS, polygenic risk score; T2D, type 2 diabetes. Created with BioRender.com

Final note: This framework combines the availability of new incretin-based agents with phenotype-guided prescribing, using genetics as a roadmap to anticipate severity and response (Figure 2).

## 4 Discussion

This review synthesizes the emerging evidence for precision obesity pharmacotherapy and highlights both its promise and current limitations. Available studies across phenotypic, genomic, transcriptomic, proteomic, metabolomic, and behavioral domains suggest clinically meaningful heterogeneity in weight-loss response to contemporary agents. However, the evidence remains limited by small sample sizes, European-ancestry enrichment, heterogeneous response definitions, variable follow-up windows, inconsistent analytic strategies, and limited external validation, which restrict reproducibility and clinical translation.

These findings complement recent guidelines and population-level syntheses showing that GLP-1RAs and dual incretin agonists now anchor contemporary pharmacologic care because of their superior efficacy and acceptable safety profiles (10). Major guidance documents also emphasize obesity as a chronic disease requiring durable management, comorbidity-informed treatment selection, and ongoing monitoring rather than short-course prescribing (87). Within this context, precision pharmacotherapy adds value by helping clinicians individualize treatment when population averages do not predict individual benefit, tolerability, persistence, or weight regain risk.

In practice, early response assessment at 12–16 weeks can serve as a structured checkpoint to guide dose optimization, treatment switching, and reinforcement of behavioral or adherence support (88). This approach is particularly useful when combined with phenotype-guided assessment. The Acosta four-phenotype model—hungry brain, hungry gut, emotional hunger, and slow-burning—offers a pragmatic framework to link dominant pathophysiological drivers with medication selection (6). However, real-world persistence must be considered, because discontinuation of weight-management medications is common and is frequently followed by weight regain and reversal of cardiometabolic benefits (89).

Genomic evidence is biologically plausible but remains less mature than phenotype-based and early-response strategies. Emerging data suggest that inherited variation may contribute to differential response to GLP-1–based therapies (82), but current pharmacogenomic findings are inconsistent and constrained by small cohorts, candidate-gene designs, and uneven ancestry representation (61, 68). Polygenic risk scores also remain far from routine clinical use because prediction does not yet translate into validated treatment selection without external validation, calibration, transparent reporting, and cross-ancestry testing, and careful assessment of potential health disparities (90, 109).

Economic sustainability and equity remain central barriers. At current prices, semaglutide and tirzepatide may not meet conventional cost-effectiveness thresholds, and broad coverage could have major budgetary implications (91). Unequal uptake may worsen obesity-related disparities if access is not addressed explicitly (92). Given current limitations in pharmacogenomic evidence, phenotype-guided pharmacotherapy may offer a more equitable interim strategy because it relies on clinical history, validated questionnaires, and feasible physiologic measures rather than high-cost genotyping (93). Future research should prioritize pragmatic, multi-ancestry studies that test integrated algorithms combining phenotyping, early response, and genomic information when appropriate, while also evaluating real-world effectiveness, persistence, affordability, and equity (94).

Finally, systems-level biomarkers, including single-cell and spatial transcriptomic approaches, may eventually refine mechanistic stratification. Evidence from other inflammatory diseases suggests that tissue architecture can reveal clinically relevant heterogeneity beyond cell abundance alone (94, 110). In obesity, adipose tissue spatial maps are emerging but remain insufficiently linked to pharmacotherapy outcomes (94). As these resources mature, AI-enabled multimodal models integrating phenotype, genomic, and spatial features may support precision pharmacotherapy, provided they are externally validated and assessed for calibration and equity (95).

## 5 Conclusion

Obesity pharmacotherapy has entered an era of high-efficacy agents, yet response variability, tolerability, adherence, and weight regain remain major clinical challenges. At present, precision pharmacotherapy is most actionable through phenotypic characteristics, including impaired satiation or “hungry brain,” reduced postprandial satiety or “hungry gut,” emotional or hedonic eating, low energy expenditure, baseline adiposity, diabetes status and glycemic profile, sex, treatment exposure, tolerability, and early weight-loss trajectory at 12–16 weeks. These features can guide realistic expectations, treatment monitoring, dose optimization, switching, and behavioral support.

Genomic and polygenic tools may eventually refine treatment selection, but current evidence remains insufficient for routine clinical use. Prospective validation, standardized response definitions, replication across diverse ancestries, and evaluation of equity and affordability are required before implementation. Therefore, the most credible current approach is a pragmatic framework combining phenotype-guided selection, early response monitoring, and adherence and tolerability management, with genomic information incorporated only once clinically validated.
